# Autoimmune Diseases Following Environmental Disasters: A Narrative Review of the Literature

**DOI:** 10.3390/healthcare12171767

**Published:** 2024-09-04

**Authors:** Alexandra Mpakosi, Vasileios Cholevas, Ioannis Tzouvelekis, Ioannis Passos, Christiana Kaliouli-Antonopoulou, Maria Mironidou-Tzouveleki

**Affiliations:** 1 Department of Microbiology, General Hospital of Nikaia “Agios Panteleimon”, 18454 Piraeus, Greece; 2 School of Medicine, University of Bologna, 40126 Bologna, Italy; 3 School of Agricultural Technology, Food Technology and Nutrition, Alexander Technological Educational Institute of Thessaloniki, 57400 Thessaloniki, Greece; tzouvelekisgiannis@yahoo.gr; 4 Surgical Department, 219, Mobile Army, Surgical Hospital, 68300 Didymoteicho, Greece; 5 Department of Immunology, General Hospital of Nikaia “Agios Panteleimon”, 18454 Piraeus, Greece; c.kalanto@gmail.com; 6 Department of Pharmacology, School of Medical, Faculty of Health Sciences, Aristotle University of Thessaloniki, 54124 Thessaloniki, Greece

**Keywords:** climate change, environmental disasters, autoimmunity, silica, particulate matter, vector-borne diseases, stress, autoantibodies

## Abstract

Environmental disasters are extreme environmental processes such as earthquakes, volcanic eruptions, landslides, tsunamis, floods, cyclones, storms, wildfires and droughts that are the consequences of the climate crisis due to human intervention in the environment. Their effects on human health have alarmed the global scientific community. Among them, autoimmune diseases, a heterogeneous group of disorders, have increased dramatically in many parts of the world, likely as a result of changes in our exposure to environmental factors. However, only a limited number of studies have attempted to discover and analyze the complex association between environmental disasters and autoimmune diseases. This narrative review has therefore tried to fill this gap. First of all, the activation pathways of autoimmunity after environmental disasters have been analyzed. It has also been shown that wildfires, earthquakes, desert dust storms and volcanic eruptions may damage human health and induce autoimmune responses to inhaled PM2.5, mainly through oxidative stress pathways, increased pro-inflammatory cytokines and epithelial barrier damage. In addition, it has been shown that heat stress, in addition to increasing pro-inflammatory cytokines, may also disrupt the intestinal barrier, thereby increasing its permeability to toxins and pathogens or inducing epigenetic changes. In addition, toxic volcanic elements may accelerate the progressive destruction of myelin, which may potentially trigger multiple sclerosis. The complex and diverse mechanisms by which vector-borne, water-, food-, and rodent-borne diseases that often follow environmental diseases may also trigger autoimmune responses have also been described. In addition, the association between post-disaster stress and the onset or worsening of autoimmune disease has been demonstrated. Given all of the above, the rapid restoration of post-disaster health services to mitigate the flare-up of autoimmune conditions is critical.

## 1. Introduction

Environmental disasters are extreme environmental phenomena of geophysical (earthquake, volcanic eruption, tsunami, etc.), hydrological (flood, landslide, etc.), meteorological (tropical storm, extreme temperature, dust storm, ice storm, wind, etc.), climatological (wildfire, drought, etc.) and extraterrestrial (geomagnetic storm, energetic particles, etc.) origins [1].

Storms, cyclones, tsunamis, wildfires, and floods can bring people into contact with microorganisms and cause them infections through injury and subsequent contamination, or through inhalation of fungal spores [2]. They can also create an environment favorable for the synthesis and increase in concentrations of particulate matter (PM) and ozone (O_3_) in the atmosphere [3]. Such disasters can also damage water and sanitation infrastructure, disrupt health care services, and affect people’s physical and mental health [4].

In addition, exposures to such extreme environmental conditions can also disrupt the human immune system by activating complex interdependent pathways. Firstly, such exposures to antigens, pollutants, toxic substances and heat stress, can damage the body’s first line of defense, the epithelial barrier, leading to inflammation, and the onset of allergic and autoimmune disorders [5,6]. Loss of biodiversity and alteration of the human microbiome, which are frequent effects of such extreme conditions, may also play a key role in triggering autoimmunity. Moreover, these stressful situations may overstimulate the innate immune system, with direct consequences on adaptive immunity potentially leading to decreased immune tolerance and also contributing to the development or exacerbation of autoimmune diseases [7]. For example, a study showed that Sjögren’s syndrome was diagnosed 1-to-3 years earlier in people living in countries exposed to extreme rainfall, wildfire, wind threats and flooding than in others who did not live in such conditions [8].

It is well established that autoimmune disorders are multifactorial diseases involving genetic and environmental factors. However, in recent decades there has been a continuous increase in their frequency, which, based on the above, could be partially attributed to the extreme environmental conditions caused by climate change [9]. Therefore, in this review, we will discuss in detail the potential association between environmental disasters and autoimmune disorders. In addition, we will try to investigate the mechanisms by which such conditions could trigger or exacerbate autoimmunity. Furthermore, we will try to highlight the critical importance of rapid post-disaster recovery of health services in mitigating the worsening of autoimmune conditions. Finally, we will attempt to provide recommendations for policy makers and healthcare providers, such as strategies for monitoring autoimmune disease outbreaks and enhancing disaster response protocols.

## 2. Methods

For this review a research methodological protocol was developed:

### 2.1. Formulation of the Research Question

For the formulation of the research question, the PICO (Population, Intervention, Comparison, Outcome) framework was used:

- Population: Disaster survivors;
- Intervention: Environmental disasters;
- Comparison: Not required for this review;
- Outcome: Autoimmune disease development.

### 2.2. Development of the Review Protocol

Research question: To conduct a review of all cases of autoimmune diseases associated with environmental disasters that have been published in the international literature.

### 2.3. Inclusion and Exclusion Criteria

This literature review included case reports and case series studies reporting on cases of autoimmune diseases associated with environmental disasters. Only studies published in the English language were included, and there were no geographical or chronological limitations. Although case reports and case series generally lack generalizability and validity and cannot establish cause-and-effect relationships, they can nevertheless provide valuable information, especially in the absence of data from randomized controlled trials (RCTs) and observational studies. For example, case reports and case series are particularly significant when they suggest an important and probable cause connection in the context of an emerging epidemic.

The exclusion criteria were review articles, systematic reviews, meta-analyses, conference proceedings, studies referring to other forms of diseases associated with environmental disasters, studies referring to autoimmune diseases associated with other environmental factors, duplicate publications of the same cases, and articles not written in English. Given the paucity of evidence in the literature, it is important to include all articles on the potential role of environmental disasters in inducing autoimmunity. Therefore, these search criteria were chosen in order to record all articles related to this topic. In addition, the researchers searched for articles that, on the one hand, were directly related to the role of environmental disasters in the pathogenesis, diagnosis and exacerbation of autoimmune diseases and, on the other hand, could contribute to new knowledge. Therefore, this review identified the most relative articles that provide useful information about the role of environmental disasters in triggering autoimmune conditions in disaster survivors. The selected articles had to cover a wide range of aspects of the topic.

Analysis of the articles included the possible mechanisms by which the environmental factors induce autoimmune responses. The reviewers also explored and highlighted the specific pathways such as oxidative stress, epithelial barrier damage and immune dysregulation by which these disasters can disrupt the immune system. In addition, the review highlighted the importance of rapidly restoring health care services to mitigate the risk outbreaks of autoimmune diseases and the necessity for improved preparedness strategies to address the long-term health impacts of disasters. Future research could use the articles in this review, integrate their findings, and offer new perspectives.

### 2.4. Search Strategy—Data Source

The search was carried out from June 2024 to July 2024 (final search date: 1 July 2024). The review was based on a comprehensive literature search at PubMed, using the following keywords: ((Environmental OR (natural) disaster) OR (wildfire) OR (earthquake) OR (dust storm) OR (heat stress) OR (climate change) OR (air pollution) OR (volcanic eruption) OR (vector-borne diseases) OR (waterborne diseases) OR (rodent-borne diseases) OR (foodborne diseases) OR (post-disaster stress)) AND ((autoimmune disease) OR (autoimmunity)).

### 2.5. Data Extraction, Disagreement Resolution and Results

In PubMed, 2235 articles were found.

Two reviewers (AM and CKA) independently decided which of these articles to select, based on the title and abstract. Conflicts were resolved through consensus and discussion between the six article reviewers (AM, VC, IT, IP, CKA and MMT). Two authors (AM and MMT) then independently assessed each remaining full-text article to decide potential inclusion in the study. Disagreement was resolved by consensus. In addition, they checked the references to include significant references from other data sources. Finally, it was decided that 200 articles met all study criteria.

## 3. Pathways Linking Climate-Induced Environmental Disasters to Autoimmune Diseases

Climate change is usually the result of an imbalance between radiation leaving and entering the Earth’s atmosphere [10]. This change is not only a phenomenon of modern times. Ever since the beginning of the world, the Earth’s climate has been constantly changing. However, since the creation of large urban settlements with large population density and large number of businesses and industries, the rate of climate change has accelerated tremendously, dramatically affecting the environment [11]. The emissions and concentrations in the atmosphere of toxic gases and pollutants from human activity, such as carbon dioxide (CO_2_), nitrogen oxides (NO_x_) and volatile organic compounds (VOCs), ammonia (NH_3_), sulfur dioxide (SO_2_) and black and organic carbon (BC, OC), also affect human health [11].

Among air pollutants, particles smaller than or equal to 2.5 µm (ultrafine and fine) are particularly dangerous, due to their ability to reach deeper into the lower airways where gas exchange takes place in the lungs [12]. In addition, particulate matter of 2.5 (PM2.5) may also present a 10,000-times greater particle-number dose per macrophage than coarse particulate matter does [13]. PM2.5 may increase airway mucosal permeability by mechanisms such as degradation of tight junction proteins, downregulation of claudin-1, occludin and E-cadherin, and disruption of barrier function. It can also disrupt structural proteins such as cytokeratin and filaggrin, and cause an increase in lysosomal membrane permeability, lipid peroxidation, and forkhead box protein P3 (FOXP3) methylation, further affecting the function of the regulatory T (Treg) cells that are responsible for immune homeostasis [14]. In addition, PM inhalation, may activate epithelial Toll-like receptors (TLRs) (TLR2 and/or TLR 4), leading to nuclear factor-κB (NF-κB) and pyrin-containing inflammasome protein 3 (NLRP3) inflammatory signaling and secretion of proinflammatory mediators, such as interleukin (IL)-1α, IL-1β, IL-6, C-X-C chemokine pattern 8 (CXCL8) and granulocyte-macrophage colony-stimulating factor (GM-CSF) [15]. Moreover, activation of the NLRP3 inflammasome by PM may induce the production of reactive oxygen species (ROSs), leading to the accumulation of activated inflammatory cells, such as macrophages, neutrophils, and dendritic cells [16]. These mechanisms of immune disruption may partially explain the findings of recent studies in which exposures to PM2.5 have been associated with higher risks of developing and/or worsening inflammatory and autoimmune diseases [17,18,19].

Climate change has also led to an increase in global temperature and a number of subsequent extreme environmental processes such as heat waves, droughts, wildfires, landslides, and melting glaciers. In addition, as the temperature rises, rivers and oceans evaporate, causing heavy storms and floods [20,21,22,23,24]. Furthermore, deglaciation appear to affect the seismic activity. It has been hypothesized that melting glaciers may lead to changes in stress distribution along major faults, possibly increasing the frequency and intensity of earthquakes. On the other hand, as the permafrost thaws, water occupies the soil’s resources, making it more unstable and prone to subsidence and landslides [25,26]. In addition, the melting of glaciers at the North and South Poles also raises the sea levels and changes the distribution of rivers [24]. Rising sea levels, along with higher sea surface temperatures, may also affect tropical storms, making them more intense and therefore more dangerous [24]. Global warming may also favor the survival and adaptation of environmental microorganisms to high temperatures [27]. Therefore, newly emerging and more heat-resistant species have appeared, such as *Candida auris*, a multi-resistant yeast, which appeared almost simultaneously around the world, probably due to climate change [28].

Climate-induced rising temperatures can also disrupt the immune system. In particular, prolonged heat wave exposure may increase levels of proinflammatory cytokines, neutrophil activation, and activation of the coagulation system [29]. Furthermore, hyperthermia may induce cell death, primarily through its effect on membrane stability, transmembrane transport protein function, and subsequent intracellular electrolyte disruption. RNA and DNA synthesis can also be disrupted by high temperatures [30]. On the other hand, anti-inflammatory pathways are activated, in an attempt to maintain homeostasis, leading to the production of interleukin 1 receptor antagonist (IL-1Ra), IL-10, soluble tumor necrosis factor (TNF) receptors and heat shock proteins. In addition, hyperthermia can damage the intestinal barrier by increasing its permeability to intestinal toxins and bacteria, triggering inflammatory responses [31].

Not only the intestinal mucosa but also the skin, and the epithelial mucous membranes of the upper and lower respiratory tract, are natural barriers between the host and the external environment, and represent the first line of defense of the human body against microorganisms, chemicals, physical injuries, environmental stress, irritants and allergens [32,33]. This is achieved by various mechanisms, including the secretion of both immunoglobulin A (IgA) and antimicrobial peptides, and the opening of epithelial barriers to remove inflammatory cells, mediators and cytokines away from the site of inflammation. In addition, epithelial cells are able to suppress inflammation with the contribution of regulatory dendritic cells, regulatory T and B cells and their anti-inflammatory cytokines [34].

Given the crucial role of the epithelial barrier in maintaining immune homeostasis, its disruption by environmental factors such as pollutants and pathogens following environmental disasters can precipitate autoimmune response [35,36,37]. For example, disruption of the intestinal barrier and subsequent dysbiosis and inflammation has been associated with systemic lupus erythematosus risk [38,39]. Increased production of pro-inflammatory cytokines, including interferon gamma (IFN-γ), TNF-α, IL-1 and IL-13 further exacerbate damage to the intestinal mucosa, as occurs in inflammatory bowel disease (IBD) [38]. It has also been found that leaky gut and proinflammatory cytokine production can damage the blood–brain barrier, leading to experimental autoimmune encephalomyelitis (EAE) in rodents, which is a similar model to multiple sclerosis in humans [40]. Leaky gut barrier has also been found to be linked to celiac disease [41]. In addition, intestinal barrier leakage facilitates the migration of inflammatory cells from the gut to the joints, potentially leading to the development of rheumatoid arthritis and ankylosing spondylitis. Leaky intestine has also been associated with the onset of type I diabetes. This has been demonstrated by mouse model experiments in which the transfer of microorganisms to pancreatic lymph nodes promoted the production of proinflammatory cytokines IL-6 and TNF, triggering the Th1/Th17-type immune response and leading to pancreatic islet inflammation [38].

On the other hand, air pollutants can cause respiratory barrier injury and damage to both the upper and lower respiratory system by triggering eosinophil proliferation and increasing IL-13 secretion in lung epithelial cells, leading to fibrosis. In addition, ozone exposure disrupts barrier integrity and leads to increased levels of intracellular ROSs, activation of NF-κB-dependent pathways, and upregulation of IL-33, IL-1α and IL-17A, causing hyperinflammation in the respiratory system, and potentially triggering asthma and chronic obstructive pulmonary disease (COPD) [42].

In addition to air pollution, other climate-induced changes in the atmosphere can make hurricanes travel more slowly, leading to heavier rainfall and deadly flooding [11,43]. In particular, frequent floods may contaminate surface and groundwater with sewage, pathogens, pesticides and chemicals, affecting freshwater supplies [44].

Rainfall and flooding are associated with the transmission of vector-borne diseases. On the one hand, they provide the necessary habitats for the larvae, while on the other hand, the high temperature affects the life cycle of the vectors and favors the growth of pathogenic microorganisms within them [45]. Outbreaks of other infectious diseases have also been associated with certain local weather conditions and global climate fluctuations. For example, high temperatures, humidity, low wind speed and diurnal temperature range (DTR) may promote the transmission of a COVID-19 (Coronavirus disease 2019) epidemic [46].

Climate change also causes more severe droughts, greater incidence of wind erosion, and increased frequency of sand and dust storms [47]. The blowing of sand and dust contributes to the Earth’s surface shaping, the erosion of rocks, and the transport of soil particles to areas even thousands of kilometers away, affecting the weather and climate, the ecosystem and the hydrological cycle [48]. Dust and sand storms may also carry plant debris, pollen, fungi and bacteria, harming human health [49].

In addition, airborne transport and deposition of microbes accompanying environmental disasters pose a further threat to the ecosystem and public health by affecting human lifestyle and diet and shaping gut microbiome composition [50].

## 4. Specific Environmental Disasters and Autoimmune Disease Risk

### 4.1. Heat Stress and Autoimmune Responses

As mentioned above, global warming and heat waves may increase heat stress and proinflammatory cytokine levels, leading to activation of both neutrophils and the coagulation system. Despite the body’s attempt to compensate for this situation with mechanisms such as the production of IL-1Ra, IL-10 receptors, soluble TNF proteins and heat shock proteins, the damage caused is often irreversible, and leads to multi-organ failure [51].

In particular, heat shock proteins belong to the alarmins, which are endogenous proteins or peptides that are released due to unscheduled cell death and which activate the immune system. Alarmins have recently been implicated in the pathogenesis of various autoimmune and immune-mediated diseases such as systemic lupus erythematosus, rheumatoid arthritis, idiopathic inflammatory myopathies, anti-neutrophil cytoplasmic antibodies (ANCA)-associated vasculitis, Behçet’s disease and cutaneous organ-specific autoimmune diseases (vitiligo, psoriasis, alopecia, and pemphigus) [51,52].

Damage to both the immune system and the epithelial barrier caused by prolonged exposure to heat stress has also been demonstrated in animal studies [53]. In mouse models, in particular, heat stress has induced epigenetic changes, with up-regulation of genes that also promote increased leukocyte migration, enrichment of monocytes, macrophages and megakaryocytes, inflammation and oxidative stress [44].

### 4.2. Wildfires and Immune System Impact

High temperatures have increased the frequency of wildfire events. On the one hand, wildfires cause burns, injuries, damaged homes, and forced migration. On the other hand, wildfire smoke can affect the air quality of areas hundreds or even thousands of miles away, contaminating it with a mixture of particles (coarse, fine, and ultrafine) of volatile organic compounds, heavy metals, and other air pollutants [54,55].

**Figure 1:**
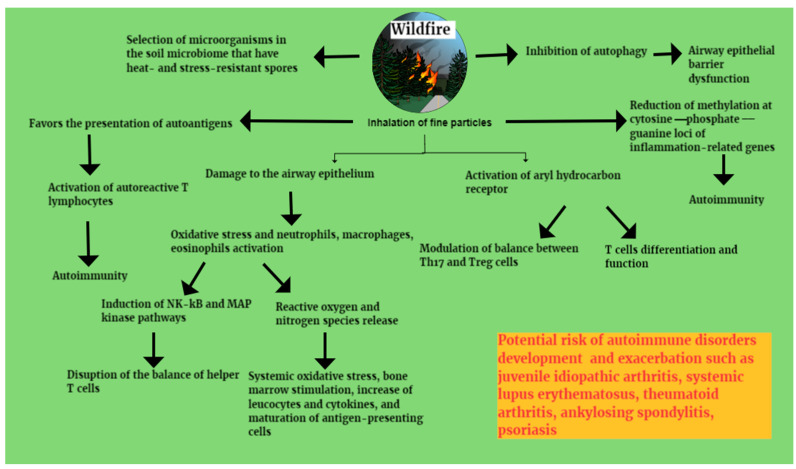
Immunological mechanisms of autoimmunity development through inhalation of fine particles from wildfire smoke and air pollution. NK-kB: nuclear factor kappa B, MAP: mitogen-activated protein, Th17: T helper 17, Treg cells: regulatory T cells.

Wildfire-smoke PM2.5 particles can be up to 10 times more toxic than those from other sources. They can generate free radicals and induce oxidative stress, with the subsequent induction of NF-κB and MAP (mitogen-activated protein) kinase pathways. NF-κB plays a critical role in regulating the survival, activation and differentiation of innate immune cells and inflammatory T cells. In particular, the activation of NF-κB by wildfire-smoke PM2.5 particles can disrupt the balance of T helper 17 (Th 17) cells [56,57,58,59]. A ligand-activated transcription factor, the aryl hydrocarbon receptor, which also contributes to differentiation and function of T cells, may also play a critical role [59]. The aryl hydrocarbon receptor is mainly expressed in Th 17, Tr1 (type 1 regulatory T cells), FOXP3 and Treg cells, followed by Th1 and Th2, and is critical in modulating the balance between Th17 and Treg cells, which play a key role in autoimmune disease [59,60]. In addition, oxidative stress caused by PM2.5 can induce bronchial-associated lymphoid tissue, associated with the production of autoantibodies on the one hand and proinflammatory cytokines on the other, which present autoantigens to autoreactive T cells, leading to autoimmunity [61,62,63,64]. Furthermore, reduced methylation at CpG (cytosine–phosphate–guanine) loci of inflammation-related genes may potentially contribute to autoimmune responses [65] (Figure 1).

Indeed, particulate-matter exposure has been associated with a higher risk of juvenile idiopathic arthritis symptoms in children under five years of age and exacerbation of other pediatric rheumatic diseases, including systemic lupus erythematosus [63,66,67]. Even short-term exposure to PM2.5 may be enough to increase the risk of the disease [68]. On the other hand, exposure of pediatric lupus patients to high PM2.5 concentrations has been associated with increased risk of nephritis, increased 24 h urine protein and leukocyturia, decreased serum C3 levels, increased double-stranded DNA (anti-dsDNA) antibodies, and increased inflammatory interleukins such as TNF-α, INF-α, IL-10 and IL-17 [69,70]. Other studies in lupus-prone mice exposed to particulate matter have confirmed worsening and acceleration of disease progression, as well as deterioration of kidney function [71].

Furthermore, Chang KH et al., Hart JE et al., and Chen H demonstrated an increased risk of rheumatoid arthritis in individuals exposed to PM2.5 [72,73,74]. Park JS et al. found a further association of 2.5 particle levels with rheumatoid arthritis, but not with other autoimmune rheumatic diseases, suggesting that the fine-particle fraction may play a role in disease pathogenesis [75]. Other studies have revealed the link between PM2.5 exposure and ankylosing spondylitis, or even psoriasis [76,77]. In addition, long-term exposure to PM has been associated with a greater risk of connective tissue diseases and inflammatory bowel disease. Also, long-term exposure to PM2.5 at levels higher than 20 μg/m^3^ has been associated with a 13% higher risk of rheumatoid arthritis and multiple sclerosis [78].

In addition to the effects of the toxic smoke in the atmosphere, the wildfire also affects the soil. Consequently, the soil microbiome and mycobiome change and adapt to wildfires by selecting microorganisms that have heat- and stress-resistant spores, properties that make them infectious at human body temperatures [2,79,80]. Thus, the exchange of aerosols between these soil microorganisms and the atmosphere may dramatically affect both ecosystem and human health. The impacts are not limited to the local level, as viable microbes and pathogenic and non-pathogenic fungal species can spread through smoke hundreds-to-thousands of miles from the fire source [81,82,83].

For example, several hospitals in California were recently reported to have increased cases of coccidioidomycosis in the months following wildfires and subsequent exposure to wildfire smoke [84]. Other environmental disasters, such as earthquakes, have also caused outbreaks of coccidioidomycosis. For example, one such outbreak was described in Ventura County, due to the dust cloud created after the January 1994 earthquake centered on Northridge, California and the subsequent landslides [85].

The spores of *Coccidioides* species are extremely resistant to adverse conditions. Chow NA et al. demonstrated that a single strain of *Coccidioides immitis* was able to colonize the soil of an area of more than 46,000 m^2^ for a time period of more than 6 years. The persistence of *Coccidioides* spp. in soils with certain chemical and microbiological properties for years has also been demonstrated in other studies [86,87]. Subsequently, high temperature, faster wind gusts, and low soil moisture appear to increase the likelihood of aerosolization of these *Coccidioides* spores (arthroconidia) in the air. High densities of localized arthroconidia on the one hand, and soil disturbances occurring during environmental disasters on the other, favor airborne transport of arthroconidia and cause dust storms [88].

Coccidioidomycosis is most commonly caused by two species, *Coccidioides immitis* and *Coccidioides posadasii*, and can develop months, or even years, after inhalation of their arthroconidia. About half of cases show no symptoms, while the rest can range from simple symptoms of the common flu syndrome to severe symptoms of life-threatening disseminated infection and meningitis in both immunocompromised and immunocompetent individuals [86].

*Coccidioides immitis* has statistically significant amino acid sequence homology with the p70 or p80 subunit of Ku, a DNA-associated autoantigen targeted by autoantibodies in patients with systemic lupus erythematosus, suggesting that when there is a genetic predisposition, infection by these fungi can trigger the initiation of autoimmunity against Ku, through molecular mimicry between fungal proteins and the Ku autoantigen [89]. MPO-ANCA (Myeloperoxidase-associated antineutrophil cytoplasmic antibody) vasculitis has also been associated with coccidiomycosis [90].

### 4.3. Earthquakes and Particulate Matter

**Figure 2:**
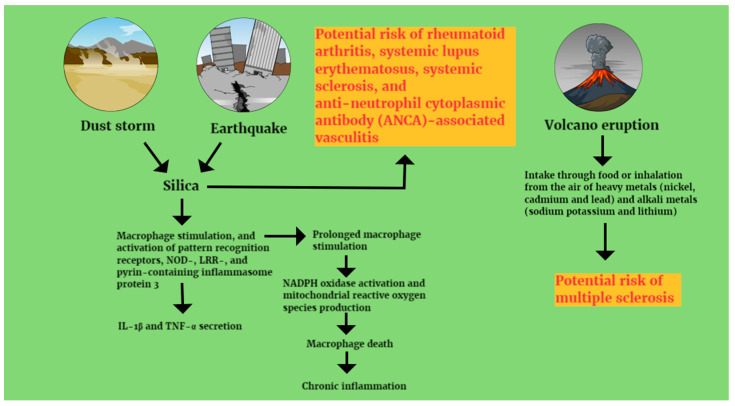
Immunological mechanisms of autoimmunity development following environmental disasters. IL-1β: interleukin 1-beta, TNF-α: tumor necrosis factor alpha, NOD: nucleotide-binding oligomerization domain, LRR: leucine-rich repeats, NADPH: nicotinamide adenine dinucleotide phosphate.

During earthquakes, PM2.5 particles may also contain dust and silica particles from collapsed buildings, car exhaust (especially diesel exhaust), and industrial and construction activities [91]. Among them, silica can stimulate macrophages, leading to the activation of PRRs (pattern recognition receptors), NOD (nucleotide-binding oligomerization domain), LRRs (leucine-rich repeats), and NLRP3, with the subsequent secretion of IL-1β, and TNF-α. A prolonged macrophage activation may trigger NADPH (nicotinamide adenine dinucleotide phosphate) oxidase activation, and mitochondrial ROS production, with eventual macrophage death and chronic inflammation. This is followed by an upregulation of airway hyperreactivity and increased levels of antinuclear antibodies [92,93] (Figure 2).

Silica exposure has been found to be associated with autoimmune diseases such as rheumatoid arthritis, systemic lupus erythematosus, systemic sclerosis, and ANCA-associated vasculitis [94,95,96,97,98,99,100,101,102,103,104].

Farquhar HJ et al. further examined the incidence of ANCA-associated vasculitis for 3 years before and for 3 years after the Christchurch earthquake of February 2011. Their study, in contrast to previous ones, did not support the hypothesis that the post-earthquake dust exposure had a causative role in the pathogenesis of ANCA-associated vasculitis. However, this may be due to differences between studies such as population density, study period, frequency of specific antibodies associated with vasculitis, or different degree of earthquake [105].

### 4.4. Desert Dust Storms and Health Risks

Climate change, with rising temperatures, drought, and desertification have also led to more frequent desert dust storms that can transport dust particles over long distances, causing serious global health problems [106,107,108,109]. For example, Saharan dust has been shown to be transported across the Atlantic Ocean and reach the Amazon, the Andes and elsewhere in the Americas, as well as moving north to Europe and the Arctic. The desert dust particles are usually composed of silicon dioxide (SiO_2_), aluminum oxide (Al_2_O_3_), iron (Fe_2_O_3_) and titanium (TiO_2_) oxides as well as calcium (CaO) and magnesium oxide (MgO) and sodium and potassium oxides (Na_2_O and K_2_O). Trace elements such as zirconium (Zr), strontium (Sr), rubidium (Rb) and rare-earth elements (REEs) are also present, depending on the composition of the source rock. Desert dust also contains large amounts of volatilized minerals (salt), microorganisms, and anthropogenic pollutants including pesticides, sulphates, nitric acid and polycyclic aromatic carbons [109]. Young children and the elderly (especially those suffering from chronic cardiopulmonary diseases), are more affected by desert dust storms. Infectious diseases such as influenza A, coccidioidomycosis, bacterial pneumonia and meningococcal meningitis, as well as non-infectious diseases such as chronic obstructive pulmonary disease, asthma, sarcoidosis, pneumoconiosis and pulmonary fibrosis, have been associated with desert dust exposure [107,108,109]. Liu TC et al. showed that the number of Taiwan hospital admissions for silicosis increased just one day after the onset of the Asian dust storm, proving that exposure and inhalation of silica contained in the desert dust can cause silicosis very quickly. The authors also argued that men are probably more susceptible to the risk of occupational silicosis, in contrast to women, who appear to be more susceptible to the environmental risk of silicosis [110] (Figure 2).

Furthermore, in in vitro tests, respirable desert dust particles have been found to be highly hemolytic and appear to induce the release of lactate dehydrogenase from alveolar macrophages. Also, the in vitro intratracheal instillation of dust has resulted in multifocal interstitial lung disease associated with aluminum silicate deposits. Moreover, desert dust can activate complement proteins from both serum and bronchoalveolar lavage, leading to chemotaxis of alveolar macrophages [108].

### 4.5. Volcanic Eruptions and Immune Triggers

During volcanic eruptions, heavy metals such as nickel, cadmium, and lead, and alkali metals such as sodium potassium and lithium are emitted. These toxic volcanic trace elements accumulate in soil, plants and groundwater used for drinking, harming both the environment and public health. The population is exposed mainly through food and water intake and inhalation from the air. Nicoletti A et al. suggested that volcanic trace elements may have played a role in the multiple sclerosis pathogenesis found at a higher prevalence and incidence in the Etna region [111] (Figure 2). The pathogenesis of the disease involves changes in the homeostasis of metals that lead to an increased level of free radicals and oxidative stress. Oxidative stress, in turn, may accelerate the progressive destruction of myelin. On the other hand, the metals may exert or exacerbate toxic effects on myelin. They may also affect immune cells and promote cytokine production. It has also been shown in animal models that endothelial surface molecules (Intercellular Adhesion Molecule 1 and Vascular Cell Adhesion Molecule 1) (ICAM-1 and VCAM-1) may be upregulated by nickel chloride (NiCl2) exposure in cultured human cells [111]. ICAM-1 and VCAM-1 are known to help leukocytes move from the circulatory compartment to tissues where there is inflammation or an immune response. For example, leukocyte adhesion to activated synovial endothelium early in the inflammatory process is the most important pathogenic mechanism in rheumatoid arthritis [112] (Table 1).

## 5. Triggering Autoimmune Diseases through Environmental Disasters

### 5.1. Vector-Borne Diseases

Heavy and frequent rainfall, flooding, and presence of stagnant water may create breeding grounds for mosquitoes, increasing their population and activity [113]. On the other hand, environmental disasters can cause the collapse of buildings and infrastructure, landslides, and the destruction of river dams, bringing changes to people’s lifestyles, and moving them to temporary shelters or outdoors at night, thus increasing their exposure to mosquitoes [114,115]. Consequently, several outbreaks of vector-borne diseases following environmental disasters such as malaria, dengue virus infection, Zika virus infection, West Nile virus infection, chikungunya virus infection, Murray Valley encephalitis, Ross River virus infection, Barmah Forest virus infection, Rift Valley fever, Japanese encephalitis etc., have been reported [116,117,118].

Several viral, bacterial, parasitic, and fungal infections, including infections following environmental disasters, have been associated with the development of autoimmune disorders by mechanisms that include molecular mimicry, bystander activation, epitope spreading, persistent infection, and polyclonal B cell activation [119,120,121,122,123,124].

For example, malaria outbreaks occur very often after environmental disasters such as earthquakes, droughts, hurricanes and floods [114,115,116,118,125,126,127,128,129,130]. Malaria leads to the production of a series of autoantibodies that target host molecules and cells, such as the phospholipid (PS). *P. falciparum*-, *P. vivax*-, *P. malariae*- and *P. knowlesi*-infected patients from different cohorts around the world have shown an inverse association between anti-PS antibodies and hemoglobin levels [131]. Malaria-induced autoimmune anemia has recently been hypothesized to be due to a TLR9/IFN-γR (TLR9/IFN-gamma receptor) synergistic activation mechanism of T-bet+ (transcription factor T-bet+) B cells. This involves both autoimmune B cells and atypical B cells, by mechanisms that are also common to many infections and autoimmune disorders such as systemic lupus erythematosus, and COVID-19. Autoimmune B cells are characterized by expression of the non-classical B cell markers CD11c (cluster of differentiation 11c) and T-bet, while atypical B cells, in addition to the expression of CD11c and T-bet, are additionally characterized by the lack of expression of CD27 and CD21 [131,132,133]. Other hypotheses for the malaria-induced autoimmune anemia may include the deposition of complement-containing immune complexes on the surface of red blood cells, resulting in a reduction in their survival time, the existence of anti-triose phosphate isomerase autoantibodies that can bind to red blood cells, promoting their solution and triggering the complement cascade, the presence of antibodies against a red-blood-cell protein, spectrin, with the subsequent development of autoimmunity through molecular mimicry, and the existence of anti-erythrocyte antibodies that cause anemia due to erythrophagocytosis or due to the reduction in red-blood-cell deformability or even their removal from circulation [134,135,136,137].

In addition, malaria has been associated with ANA (antinuclear antibody) and anti-ssDNA antibody (anti-single-stranded DNA antibody) positivity in both Caucasians, who had traveled to tropical countries, and local residents of malaria-endemic areas [138]. Furthermore, in another study of a Ugandan pediatric cohort with severe *P. falciparum* malaria, anti-dsDNA antibodies were associated with acute kidney injury, high morbidity and mortality [139]. It has also been hypothesized that malaria may trigger or exacerbate an underlying systemic lupus erythematosus, already subclinical, possibly due to the presence of autoantigens from the damaged tissues [138]. Autoantibodies against brain-associated antigens have also been reported in patients with *P. falciparum* cerebral malaria, but their significance is still unclear [137].

Another example is dengue, which is one of the most common vector-borne viral diseases following environmental disasters [118,126,140]. The clinical manifestations of the disease are probably due to molecular mimicry of dengue virus proteins such as DENV NS1 (dengue virus non-structural 1), DENV prM (dengue virus pre-membrane) and E (envelope), with autoantigens such as coagulation factors, platelets and endothelial cells. The resulting autoantibodies may lead to hemostasis, thrombocytopenia, hepatic endothelial dysfunction and pemphigus [141,142,143,144,145]. Dengue infection has also been associated with several autoimmune disorders such as Reiter’s syndrome, multiple sclerosis, myasthenia gravis, autoimmune encephalomyelitis, systemic vasculitis, systemic lupus erythematosus, and primary adrenocortical insufficiency [146,147,148]. However, Shih HI et al. have suggested that dengue infection may only be associated with an increased risk of autoimmune encephalomyelitis, and no other autoimmune disease [149].

Zika virus infection is another common vector-borne disease following environmental disasters [150,151,152,153,154]. Zika virus has been associated with Guillain–Barré syndrome and idiopathic thrombocytopenic purpura [155,156,157,158,159]. França LC et al. recently found a high identity between Zika virus nonstructural protein 5 (NS5) and myelin proteolipid protein, which is an autoantigen of multiple sclerosis, suggesting that molecular mimicry may be possible between the Zika virus and inflammatory demyelinating disorders of the central nervous system [160].

West Nile is another mosquito-borne flavivirus infection that has also been associated with environmental disasters [116,161,162]. The West Nile virus may trigger autoimmune encephalitis related to the anti-glycine receptor antibodies or amphiphysin antibodies [163,164]. Associations between the West Nile virus and myasthenia gravis, possibly through molecular mimicry between virus antigens and acetylcholine receptor antibodies, and between West Nile and Guillain–Barré syndrome, have also been suggested [165,166,167,168].

Furthermore, Chikungunya virus infection may induce autoantibodies and trigger rheumatic diseases, such as rheumatoid arthritis, spondylarthritis and Sjögren syndrome [169,170,171,172,173].

Another infection following environmental disasters is Leishmaniasis, which is one of the most commonly emerging parasitic diseases in recent years. It is caused by *Leishmania* spp. transmitted by the sand fly. Habitat alteration following environmental disasters may affect the vector abundance and activity, increasing the transmission of the infection [174,175,176,177]. During visceral leishmaniasis, elevated autoantibodies such as ANA, ANCA, anti-mitochondrial M2 antibodies (AMA-M2), anti-liver cytosol specific type 1 antibodies (anti-LC1), anti-liver/kidney microsomal type 1 antibodies (anti-LKM1), anti-centromere protein-B antibodies (anti-CENP-B), anti-Sjögren’s syndrome type A antibodies (anti-SSA), anti-Sjögren’s syndrome type B antibodies (anti-SSB), anti-Jo-1 antibodies, anti-dsDNA and rheumatoid factor (RF), are common [178,179] (Table 2).

### 5.2. Waterborne and Foodborne Diseases

Waterborne and foodborne diseases are very common after environmental disasters [180,181,182]. Heavy rainfall and cyclonic storm may cause flooding of the infrastructure of the water supply system, sewage overflow, contamination of environmental sources and drinking water, and also irrigation water used in crops, affecting the transmission of pathogens [183,184]. Outbreaks among the displaced population in emergency shelters are usually associated with ingestion of water or food contaminated with microorganisms from human or animal feces, shared water containers and cooking utensils, lack of sanitation, and crowding [182,185]. Hepatitis A and E, cholera, shigellosis, salmonellosis, cryptosporidiosis, giardiasis, and rotavirus infection have been reported more frequently [184,186,187,188,189,190].

Foodborne infections can cause long-term problems in many organ systems, including irritable bowel syndrome, inflammatory bowel disease, reactive arthritis, hemolytic uremic syndrome, chronic kidney disease, and Guillain–Barré syndrome [191]. Tsuboi H et al. reported that the annual incidence of Guillain–Barré syndrome and Fisher syndrome had increased significantly in the year following the 2011 Great East Japan Earthquake, mainly due to post-earthquake gastrointestinal infections [192].

Both *Salmonella* and *Shigella* have been implicated in the development of inflammatory bowel disease, including Crohn’s disease and ulcerative colitis, reactive arthritis and Reiter’s syndrome [193,194,195,196,197,198].

In particular, reactive arthritis is associated with the presence of HLA-B27 (Human Leucocyte Antigen-B27). It seems that *Salmonella* alters cellular localization within HLA-B27-expressing epithelial cells. Both the tissue damage and dysregulation of the host’s adaptive immune response may subsequently trigger autoimmunity [199]. Indeed, infected MyD88−/− (Myeloid differentiation primary response 88) mice were found to have high serum titers of not only anti-*Salmonella* antibodies but also anti-dsDNA, autoantibodies directed against thyroglobulin, and RF, positive nuclear staining with HEp-2 cells (human epithelial-2 cells), and deposition of immune complexes in the kidneys [200]. In addition, Tu TY et al. suggested that non-typhoidal *Salmonella* infection may trigger systemic lupus erythematosus, through the production of proinflammatory cytokines [201].

The parasites *Giardia* and *Cryptosporidium* have also been implicated in reactive arthritis and Crohn’s disease [198,202,203]. Moreover, a transient increase in anti-tissue transglutaminase (tTG) and anti-endomysial antibodies (EMAs) has also been reported during or shortly after *Giardia* infection [204].

Other common infections following environmental disasters are Hepatitis A and E. Hepatitis A and E viruses may possibly trigger autoimmune hepatitis [205,206,207,208]. However, few patients with acute hepatitis E may develop hepatitis-specific autoimmune autoantibodies without subsequently developing autoimmune hepatitis [209]. The Hepatitis E virus has also been implicated in triggering reactive arthritis, Guillain–Barré syndrome, cryoglobulin production, autoimmune thyroiditis, cutaneous necrotizing small-vessel vasculitis (CNSVV) and Henoch–Schönlein purpura [210,211,212,213,214,215,216].

Guillain–Barré syndrome, rheumatoid arthritis, Still’s syndrome, Henoch–Schönlein purpura, autoimmune hemolytic anemia, antiphospholipid syndrome, systemic lupus erythematosus and cryoglobulinemic vasculitis have also been associated with Hepatitis A virus. Induced immune response and antigenic molecular mimicry have been implicated as triggers of autoimmunity [217,218].

*Vibrio cholerae* cholera toxin B (CTB) has been found to trigger disease progression in lupus-prone mice by enhancing T cell lipid raft aggregation [219].

Rotavirus infection has also been associated with autoimmune diseases such as celiac disease, Henoch–Schönlein purpura, type 1 diabetes, uveitis, pemphigus vulgaris, myasthenia gravis, and biliary atresia, probably induced by molecular mimicry of rotavirus peptides [220,221,222,223,224,225,226,227,228,229,230].

Schistosomiasis usually follows flooding and the subsequent public health, sanitation and drinking-water supply problems [231,232,233,234,235]. Schistosomiasis has been associated with hepatic fibrosis [236,237]. In addition, children with schistosomiasis have been found to present a high prevalence of islet cell-antibody seropositivity and reduced early insulin release in response in the intravenous glucose load, suggesting a long-term risk for glucose intolerance and the development of diabetes [238]. ANA positivity has also been reported in *S. mansoni*, *S. haematobium* and *S. japonicum* infections [239]. In addition, anti-collagen V autoantibodies have been found to be higher in patients with schistosomiasis than in healthy individuals. Autoantibodies against collagen V are also common in recipients who develop bronchiolitis obstructive syndrome (BOS) after lung transplantation, before the development of fibrosis [240].

### 5.3. Rodent-Borne Diseases

Extreme rainfall and flooding, high temperatures, overcrowding, poor sanitation, inadequate health care systems, poverty, and a high presence of rats and other animal reservoirs have been associated with outbreaks of leptospirosis [241,242,243,244,245,246,247,248].

Lip L32 (leptospiral lipoprotein 32), one of the *Leptospira* outer-membrane proteins, may activate the Toll-like receptor 2 pathway, triggering innate responses, tubular damage and autoimmunity. Leptospiral immunoglobulin-like protein A (LigA) is a potential target for *Leptospira*-specific cell-mediated immunity [249,250,251,252]. Indeed, leptospirosis has been associated with several autoimmune diseases, including reactive arthritis and autoimmune hemolytic anemia, and has been considered as a possible trigger of systemic lupus erythematosus with positivity of ANA, anti-dsDNA, anti-Ro/La, anti-Smith and anti-ribosomal P antibodies [253,254,255,256]. Autoantibodies causing pulmonary hemorrhage syndrome and uveitis and anti-cardiolipin antibodies may also be found in acute leptospirosis [257,258,259,260]. *Leptospira* can also cause autoimmune basal ganglia encephalitis in children, due to antibodies against cell surface dopamine-2 receptors [261].

The risk of developing autoimmune disease may persist for years after acute leptospirosis, due to the ability of *Leptospira* to colonize and replicate in renal tubules, triggering autoimmunity and further deposition of complement and immunoglobulin in the human alveolar basement membrane [262,263,264,265]. Both natural killer (NK) cells and memory T cells are likely involved in this [266].

Hanta virus outbreaks have also been described, following environmental disasters such as floods [267]. Hantaviruses are RNA viruses, and belong to the *Hantaviridae* family. Humans usually become infected after inhaling aerosols from the secretions of infected rodents. Two clinical syndromes are associated with hantavirus infection in humans: hemorrhagic fever with renal syndrome (HFRS) and hantavirus pulmonary syndrome (HPS). In addition, two main immunopathological mechanisms are involved in hantavirus infection. On the one hand, the virus can induce strong T cell response, which can affect endothelial cell function by secreting excessive amounts of cytokines. On the other hand, if adequate T-cell responses are not elicited, viral clearance is delayed, leading to prolonged inflammation, which may also affect endothelial cell function by other mechanisms such as increasing the sensitivity of infected endothelial cells to vascular endothelial growth factor [268]. It has been found that hantavirus may trigger acute exacerbation of autoimmune liver disease, Guillain–Barré syndrome, autoimmune polyendocrinopathy and hypophysitis, and Graves’ disease [269,270,271,272] (Table 3).

## 6. Post-Disaster Stress and Autoimmune Disease Exacerbation

The hypothalamic–pituitary–adrenal (HPA) axis and the sympathetic nervous system (SNS) are responsible for the stress response. Neuroendocrine and autonomic responses are mediated by hormones including epinephrine, norepinephrine, corticotropin-releasing hormone (CRH), adrenocorticotropic hormone (ACTH), and glucocorticoids. Glucocorticoids, in particular, seem to play an important role in immune regulation, mainly due to their immunosuppressive effects during inflammation [273]. In addition, individuals with post-traumatic stress disorder (PTSD) have been found to have excessively low cortisol levels, particularly in the context of early-life trauma exposure. Therefore, it stands to reason that long-term lower cortisol levels could cause increased production of proinflammatory cytokines that may exacerbate autoimmune responses [274]. In addition, stressful events and subsequent dysfunctional interactions in the brain–gut axis may contribute to the pathophysiology of diseases such as irritable bowel syndrome and inflammatory bowel disease. Furthermore, psychological stress may damage the intestinal barrier, leading to microbiοme dysbiosis, intestinal hyperpermeability, intestinal inflammation, and, potentially, systemic inflammation and autoimmune disease [275]. For example, type 1 diabetes is associated with dysbiosis, intestinal hyperpermeability, increased local and systemic IL-17 secretion, and increased myeloperoxidase in the gut. In addition, systemic lupus erythematosus is associated with dysbiosis and, on the one hand, increased intestinal secretion of IL-17 and IL-22 from T cells and IFN-α and IFN-β from dendritic cells, and, on the other hand, systemic secretion of IL-6 and TNF-α by monocytes and macrophages [9].

Environmental disasters are considered particularly stressful events that can disrupt immune regulation, potentially initiating or exacerbating autoimmune conditions. After such hazards, people fear for their lives, suffer from lack of food, water, sanitation, and electricity, and are saddened by seeing their homes damaged and a friend or family member lost or displaced [276,277]. Post-disaster stress predisposes survivors to depression and chronic stress, low life satisfaction, and pessimism. All these negative emotions can particularly threaten both the short-term and long-term physical and mental health of children, even pushing them into bad addictions such as drinking alcohol or using drugs [278,279,280,281]. In addition, post-disaster stress may also predispose the elderly to cardiovascular events such as acute myocardial infarction, cardiomyopathy, heart failure, stroke, arrhythmias, increased blood pressure and pulmonary embolism, and make them vulnerable to insomnia and depression [282,283,284].

Indeed, several retrospective studies have shown the association between post-disaster stress and autoimmune disease onset or exacerbation [285,286]. Watanabe H et al. reported a case of a patient with Sjögren’s syndrome who subsequently developed systemic sclerosis and Behçet’s disease, due to serious stress after an earthquake disaster and the death of her father [287]. Oral pemphigus vulgaris following the 2010 Chile earthquake, probably due to post-disaster anxiety and depression, has also been reported [288]. Recurrences of ulcerative colitis and Crohn’s disease have also been associated with stress following environmental disasters. Miyazawa T et al. have suggested that, immediately after the earthquake, relapses may be due to hopelessness and survival anxiety. Conversely, many months after the disaster, recovery in patients’ lives and reduction in environmental stressors are associated with lower relapse rates [4]. Post-disaster stress may also affect rheumatoid arthritis activity and exacerbate systemic lupus erythematosus [289,290].

## 7. Public Health Implications

**Figure 3:**
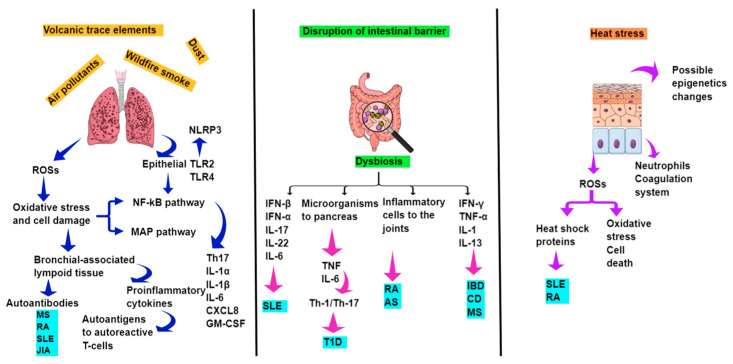
Pathways linking environmental disasters to autoimmune diseases. ROSs: Reactive oxygen species, NLRP3: Pyrin-containing inflammasome protein 3, TLR: Toll-like receptor, NF-kB: Nuclear factor kappa B, MAP: Mitogen-activated protein, Th-17: T helper-17, IL: Interleukin, CXCL8: Chemokine pattern 8, GM-CSF: Granulocyte-macrophage colony-stimulating factor, IFN: Interferon, TNF: Tumor necrosis factor, MS: Multiple sclerosis, RA: Rheumatoid arthritis, SLE: Systemic lupus erythematosus, JIA: Juvenile idiopathic arthritis, T1D: Type 1 diabetes, AS: Ankylosing spondylitis, IBD: Inflammatory bowel disease, CD: Celiac disease.

As mentioned above, inhalation of air pollutants, wildfire smoke, dust, and volcanic trace elements can damage the mucous membrane of the airways and, through complex mechanisms, can lead to the production of reactive oxygen species, oxidative stress, cell damage, induction of bronchial-related lymphoid tissue and production of autoantibodies. Heat stress can also disrupt the immune system by increasing proinflammatory cytokines and leading to the production of reactive oxygen species, heat shock proteins, oxidative stress and cell death, as well. Hyperthermia can also damage the intestinal barrier by increasing its permeability to intestinal toxins and bacteria, triggering inflammatory and autoimmune responses (Figure 3). In addition, infections following environmental disasters can also lead to autoimmune diseases through mechanisms such as molecular mimicry, bystander activation, epitope spreading, persistent infection, and polyclonal B cell activation. Consequently, public health policy must intervene with specific measures to mitigate these effects of environmental disasters on human health.

Disaster preparedness plans should take into account the health needs of populations affected by disasters. The immediate health effects are mainly related to the overcrowding of large numbers of survivors, and insufficient access to safe water and sewage facilities. Ensuring the supply of safe drinking water, and the planning of settlements with sufficient access to water supply and drainage needs and the minimum space requirements per person, are therefore important measures to be taken. In addition, access to primary care is critical for disease prevention, early diagnosis and treatment. Therefore, the post-disaster rapid restoration of affected health services is essential [291].

Of particular importance is the rapid post-disaster restoration of health services, to mitigate the exacerbacion of autoimmune conditions. Ensuring the availability and implementation of treatment protocols for patients with chronic diseases such as autoimmune disorders is imperative. However, in environmental disasters there is usually a shortage of drugs, with tragic consequences for the treatment of both acute and chronic diseases. Moreover, in such chaotic conditions, there is no care or control regarding the receiving of donated drugs, while those arriving from foreign countries may be unknown to doctors, with different doses compared to local ones. Consequently, many patients with autoimmune diseases remain without their medication.

Furthermore, to maintain public health and provide healthcare services, other agencies are also involved, including the police for security, the Water and Sewerage Agency for providing safe drinking water, and the Department of Roads and Transport for opening roads for easy and safe transportation of both patients and biological samples. In particular, ensuring sampling and transport of materials for both early detection of a possible autoimmune disease exacerbation and rapid detection of new autoimmune disease cases is essential to ensure prompt control. Although coordination between these agencies is necessary, it is often impossible, due to the general chaos caused by environmental disasters [292].

Health personnel should be familiar with diseases caused by environmental disasters, and with how to respond to them. Consequently, their continuous training in organizations is particularly useful. Moreover, healthcare providers should be trained in both the early detection and proper management of autoimmune conditions. In addition, policymakers should develop strategies to monitor autoimmune disease flare-ups. They should also develop measures to support the mental health of vulnerable survivors, such as the establishment and operation of psychological support centers, even long after the disaster [292]. Disaster response teams could also contribute to this by alleviating the sense of fear and training people to manage post-disaster stress. They could also encourage patients to seek early treatment in the case of a possible worsening of their illness [293]. Psychologists, psychiatrists, and social workers should be available for the psychological and emotional support of affected people, including women, children, and the elderly.

Adapting to climate change, raising awareness, and communicating risks to autoimmune disease patients living in countries affected by extreme environmental conditions are also important. Also, policymakers need to design climate-crisis mitigation and adaptation strategies and strengthen disaster response protocols, such as implementing education programs, monitoring hazards, developing early-warning systems using science and technology, building protective infrastructure, and developing resilient health systems [293]. For example, the installation of a telemedicine system is one of the modern technologies that could contribute to the treatment of patients with chronic diseases when the roads are closed due to the disaster. Such modern technologies should be available in all medical services for faster disaster response [292].

As mentioned above, air pollutants, dust, wildfire smoke, and volcanic trace elements all lead through similar, complex pathways to trigger and/or exacerbate autoimmune diseases. Indeed, each 10 μg/m^3^ increase in PM10 has been associated with a 7% increased risk of developing autoimmune disease, while exposure to PM10 > 30 μg/m^3^ and PM2.5 > 20 μg/m^3^ has been associated with a 12% and 13% higher risk of autoimmune disease, respectively [294]. Consequently, decisive measures are needed to limit them, for example by regulating agricultural or industrial activities, fossil fuels, waste management, domestic energy and transport. Although there is a lack of evidence on the influence of improving air quality on autoimmune diseases, data on other immune-mediated conditions, such as asthma, have suggested encouraging effects. For example, in California, measures to reduce PM2.5 and NO_2_ between 1993 and 2014 succeeded in reducing the risk of asthma in children by 20%. In Japan, 2001 legislation to limit transport-related emissions led to reductions in PM2.5 and NO_2_ and a lower (0.6–1.1%) prevalence of childhood asthma. Moreover, in Utah Valley, hospital admissions for childhood asthma were cut in half and PM2.5 was reduced, due to the closure of a steel mill for 13 months. Beneficial interventions have also been made during the Olympic Games, such as in Atlanta in 1996, where a 17-day “alternative transport strategy” resulted in a 23% reduction in morning rush hour traffic, a 42% reduction in children seeking medical care, and a 19% reduction in hospitalizations for asthma. Even the Beijing Olympics in 2008, the government instituted factory emission and travel restrictions that reduced pollutant concentrations by up to 62%, improving lung function in patients with asthma [294].

The burning of garbage and dry grasses in the countryside can sometimes reach uncontrolled proportions and lead to a wildfire, which is also a major source of air pollution. It is therefore mandatory to avoid the use of a grill, welding, and the use of a wheel or other tools that may cause sparks in outdoor areas, in the forest, or in areas where there are dry grasses and branches. Moreover, throwing lighted cigarettes, matches or garbage, and using flares in the forest, also favors the occurrence of wildfire. Deforestation of dry grass areas and their removal is therefore required in outdoor public gathering areas to avoid starting and spreading wildifires. Proper fencing for outdoor or semi-outdoor storage areas of flammable materials and particularly their removal from sources of heat emission, especially from areas where open flames are used and sparks are caused, as well as diligent maintenance of electrical installations, are also fire prevention practices. Governments can help in these directions by issuing ordinances, creating incentives, and educating the public on best practices. Public health organizations should also provide people with guidance on how to protect themselves from wildfire smoke inhalation, including moving away from the wildfire area or staying indoors by keeping windows and doors closed and using air conditioning which has indoor air recirculation and well-maintained filters, avoiding outdoor activities and drinking plenty of water and fluids. Furthermore, people suffering from wildfires could benefit from clean-air shelters that reduce PM exposure and indoor air pollution by adding high-efficiency air filters [295]. Similarly, medical personnel should inform the public of the long-term health risks of inhaling desert dust and particulate matter from earthquakes and volcanoes, and recommend the use of protective masks, the avoidance of outdoor activities, and good indoor ventilation.

## 8. Limitations

This study has several limitations. Firstly, there is a lack of evidence, as most of the disaster publications are limited to studies of mortality and/or morbidity, mainly due to infections and other acute health problems. Few studies have highlighted the long-term health problems that can be caused by extreme environmental conditions. Furthermore, the collapse of health facilities and health care systems and the abrupt cessation of surveillance and health programs, which often occur after a disaster, do not help to properly record, and with the surveillance of, chronic health problems. In addition, the extended delay times of restoring health services contribute to inaccurate surveillance. On the other hand, surveillance does not usually include the stressor, and may lead to underdetection of stress-related autoimmune disorders. We hypothesize, therefore, that the problem may be much larger and that there may be more cases of autoimmune diseases that were actually triggered by environmental disasters, but were likely not reported.

Other limitations are the limitation of analyses for autoimmune diseases and the different strategies between countries for reliable diagnosis and, also, the lack of patient data such as educational and socioeconomic status, smoking, occupation and other relevant variables, which may affect the prevalence of autoimmune disorders. Furthermore, there are no standardized registries for all autoimmune diseases worldwide. Moreover, autoimmune disorders are caused by many factors (genetic, epigenetic and environmental) that could not be controlled or reported in the original sources and were therefore not considered in this review. The possible role of other non-measurable factors should, therefore, be considered in future studies. Also, additional research should include more comprehensive, longitudinal studies of stress-related autoimmune diseases caused by extreme environmental events in higher-risk groups such as children or pregnant women, to better elucidate the triggering pathways of autoimmunity.

## 9. Conclusions

In our opinion, the contribution of this study is important. We provided a comprehensive review of the potential relationship between environmental factors and autoimmune diseases, which is an important and timely topic. In addition, we demonstrated the mechanisms linking environmental disasters to autoimmune diseases, including inhalation of air pollutants, particularly PM2.5, epithelial barrier disruption, dysregulation of immune responses to specific environmental disasters, infections and post-disaster stress. Furthermore, we highlighted the critical importance of rapid recovery of health services after a disaster in mitigating the worsening of autoimmune conditions and provided recommendations for policy makers and health care providers on disaster response strategies and autoimmune disease surveillance.

The ever-increasing frequency and intensity of environmental disasters calls for rapid elucidation of their role in inducing autoimmunity and design of innovative research protocols. The autoimmunity-triggering factors discussed above can therefore be used in the future development of new study protocols. Future research should elucidate the role of specific environmental factors and the complex mechanisms by which they can induce autoimmune conditions, the role of the immune system in extreme environmental hazards, and the role of predisposing factors and vulnerability of different socio-economic groups in environmental disasters. We believe that long-term health monitoring studies of people living in areas affected by extreme environmental conditions would be very useful, as we could more accurately control the risk of developing autoimmune diseases. Moreover, additional research should be conducted with modern biological and epidemiological tools and cover different environments in different global settings. Future research also needs to be collaborative and interdisciplinary, with the broad application of new data science techniques, to better understand the role of environmental disasters in autoimmune diseases. For example, a better collaboration between physicians and environmental scientists to jointly investigate specific environmental factors and chemical compositions of particles and dust that may be harmful to human health could yield valuable conclusions that will contribute to the formulation of specific public health policy measures.

Given the complex mechanisms and multidimensional parameters that characterize autoimmune diseases, proving the relationship between them and environmental disasters is a major challenge for researchers. Future research protocols should therefore include vulnerable populations, which will be studied over a long period of time, taking into account all genetic, epigenetic and environmental factors, in order to draw correct conclusions. Researchers should also consider post-disaster survivors’ multiple concurrent exposures to specific environmental factors and their cumulative lifetime effects, when drawing conclusions. Observational studies should also explore predisposing factors before birth, during labor, and during the first years of life, which should also be taken into account. Furthermore, a standardized research protocol to quantify exposure to specific environmental factors and investigate the association between them and autoimmune diseases, based on mathematical models and statistical analyses, would ensure reliability and comparability between studies. Big-data monitoring methods could also evaluate, using sciences such as statistics and bioinformatics, air quality or the composition of pollutants from wildfires and earthquakes and their effects on human health. The results of these studies will contribute to the development of measures to prevent or manage autoimmune diseases after environmental disasters.

## Figures and Tables

**Table 1:** Specific Environmental Disasters and Environmental Factors and Their Associated Autoimmune Responses and Diseases.

Environmental Disasters	Environmental Factors	Biological Mechanisms	Autoimmune Responses/Diseases	References
Heat wave	High temperature	Heat stress Increased pro-inflammatory cytokines Damage to epithelial barrier Epigenetic changes Heat shock proteins	Systemic lupus erythematosus, Rheumatoid arthritis, Idiopathic inflammatory myopathies, ANCA-associated Vasculitis, Behçet’s disease, cutaneous organ-specific autoimmune diseases (Vitiligo, Psoriasis, Alopecia, Pemphigus)	[51,52]
Wildfire	PM2.5 particles	Oxidative stress Damage to epithelial barrier Increased pro-inflammatory cytokines	Juvenile idiopathic arthritis, Systemic lupus erythematosus, Rheumatoid arthritis, Ankylosing spondylitis, Psoriasis, connective tissue diseases, Inflammatory Bowel disease, Multiple sclerosis	[67,68,75,76,77,78]
	Soil microbiome disruption	Inhalation of heat- and stress-resistant spores such as *Coccidioides* spores	Autoantibodies against Ku autoantigen MPO-ANCA Vasculitis	[89,90]
Earthquake	PM2.5 particles containing dust and silica	Oxidative stress Damage to epithelial barrier Macrophage death Chronic inflammation	Antinuclear antibodies Rheumatoid arthritis, Systemic lupus erythematosus, Systemic sclerosis, ANCA-associated Vasculitis	[92,93,94,95,96,98,99]
Desert Dust Storm	Dust particles containing silicon dioxide (SiO_2_), aluminum oxide (Al_2_O_3_), iron (Fe_2_O_3_) and titanium (TiO_2_) oxides	Oxidative stress Damage to epithelial barrier Activation of complement proteins Chemotaxis of alveolar macrophages and inflammation	Sarcoidosis, Pneumoconiosis, Pulmonary fibrosis, Silicosis	[107,108,109,110]
Volcanic Eruption	Toxic volcanic trace elements containing heavy and alkali metals	Oxidative stress Accelerating the progressive destruction of myelin	Multiple sclerosis	[111]

ANCA: anti-neutrophil cytoplasmic antibody, MPO: Myeloperoxidase, PM: Particulate matter.

**Table 2:** Vector-Borne Diseases and Their Associated Autoimmune Responses and Diseases.

Diseases	Associated Autoimmune Responses and Diseases	References
Malaria	Autoimmune anemia	[131,132,133,134,135,136,137]
	Anti-phospholipid antibodies	[131]
	Systemic lupus erythematosus	[138]
	ANA antibodies	[138]
	Anti-dsDNA antibodies	[139]
	Anti-ssDNA antibodies	[138]
Dengue	Myasthenia Gravis	[146,147]
	Autoantibodies against coagulation factors, platelets and endothelial cells, pemphigus	[141,142,143,144,145]
	Multiple sclerosis	[146,147]
	Reiter’s syndrome	[146,147]
	Autoimmune encephalomyelitis	[149]
	Systemic vasculitis	[148]
	Systemic lupus erythematosus	[146,147]
	Primary adrenocortical insufficiency	[146,147]
Zika	Guillain–Barré syndrome	[155,156,159]
	Idiopathic thrombocyto-penic purpura	[157,158]
	Multiple sclerosis	[160]
West Nile	Autoimmune encephalitis	[163,164]
	Myasthenia gravis	[165]
	Guillain–Barré syndrome	[166,167,168]
Chikungunya	Rheumatoid arthritis	[169,170,171]
	Sjögren’s syndrome	[172,173]
Leishmaniasis	ANA, ANCA, AMA-M2, anti-LC1, anti-LKM1, anti CENP-B, anti-SSA, anti-SSB, anti-Jo1, anti-dsDNA, and RF positivity	[178,179]

ANA: antinuclear antibodies, ANCA: anti-neutrophil cytoplasmic antibodies, AMA-M2: anti-mitochondrial M2 antibodies, anti-LC1: anti-liver cytosol specific type 1 antibodies, anti-LKM1: anti-liver/kidney microsomal type 1 antibodies, anti CENP-B: anti-centromere protein-B antibodies, anti-SSA: anti-Sjögren’s syndrome type A antibodies, anti-SSB: anti-Sjögren’s syndrome type B antibodies, anti-dsDNA: anti-double-stranded DNA antibodies, anti-ssDNA: anti-single-stranded DNA antibodies, RF: rheumatoid factor.

**Table 3:** Waterborne, Foodborne and Rodent-Borne Diseases and Their Associated Autoimmune Responses and Diseases.

Diseases	Associated Autoimmune Responses and Diseases	References
Salmonellosis	Ulcerative colitis	[195,196]
	Crohn’s disease	[195,196]
	Reactive arthritis	[194]
	Reiter’s syndrome	[197]
	SLE	[201]
	HLA-B27	[199]
	Anti-dsDNA, antithyroglobulin, RF and nuclear staining with HEp-2 cell positivity	[200]
Shigellosis	Ulcerative colitis	[193,198]
	Crohn’s disease	[193,198]
	HLA-B27	[193,198]
Giardiasis	Crohn’s disease	[202]
	Reactive arthritis	[203]
	tTG and EMA antibody positivity	[204]
Cryptosporidiosis	Crohn’s disease	[202]
	Reactive arthritis	[203]
Hepatitis A	Guillain–Barré syndrome	[217,218]
	RA	[217,218]
	Henoch–Schönlein purpura	[217,218]
	Still’s syndrome	[217,218]
	Autoimmune hepatitis	[205,206,207,208]
	Autoimmune anemia	[218]
	Antiphospholipid syndrome	[217,218]
	SLE	[217,218]
	Cryoglobulinemic vasculitis	[217,218]
Hepatitis E	Guillain–Barré syndrome	[210,211]
	Reactive arthritis	[214]
	Henoch–Schönlein purpura	[210,211]
	Autoimmune thyroiditis	[212]
	Autoimmune hepatitis	[209]
	Cryoglobulinemic vasculitis	[215]
	Cutaneous necrotizing small-vessel vasculitis	[213]
Cholera	SLE-progression trigger	[219]
Rota infection	Henoch–Schönlein purpura	[224]
	Uveitis	[226]
	Pemphigus vulgaris	[227]
	Myasthenia Gravis	[228]
	Biliary atresia	[229]
	Type 1 diabetes	[221]
	Celiac disease	[220,222]
Schistosomiasis	Hepatic fibrosis	[236,237]
	ANA antibody positivity	[239]
	Islet-cell antibody seropositivity	[238]
	Anti-collagen V autoantibodies	[240]
Leptospirosis	Reactive arthritis	[253]
	Autoimmune anemia	[256]
	SLE	[254]
	Uveitis	[258,259]
	ANA, anti-dsDNA, anti-SSA/SSB, anti-Sm, anti-ribosomal P, anti-cardiolipin positivity	[255,260]
	Autoimmune Basal Ganglia Encephalitis	[261]
Hantavirus infection	Exacerbation of autoimmune liver disease	[271]
	Guillain–Barré syndrome	[272]
	Autoimmune polyendocrinopathy and hypophysitis	[270]
	Graves’ disease	[269]

SLE: systemic lupus erythematosus, RA: Rheumatoid arthritis, ANA: antinuclear antibodies, HLA-B27: Human Leucocyte Antigen-B27, tTG: serum anti-tissue transglutaminase, EMA: anti-endomysium antibodies, anti-dsDNA: anti-double-stranded DNA antibodies, anti-Sm: anti-Smith antibodies, RF: rheumatoid factor, anti-SSA: anti-Sjögren’s syndrome type A antibodies, anti-SSB: anti-Sjögren’s syndrome type B antibodies, anti-Sm: anti-Smith, HEp-2: human epithelial cells.

## Data Availability

Data are contained within the article.
